# First report of *Cytauxzoon* sp. infection in domestic cats in Switzerland: natural and transfusion-transmitted infections

**DOI:** 10.1186/s13071-018-2728-5

**Published:** 2018-05-10

**Authors:** Alice Nentwig, Marina L. Meli, Johanna Schrack, Iris M. Reichler, Barbara Riond, Corinne Gloor, Judith Howard, Regina Hofmann-Lehmann, Barbara Willi

**Affiliations:** 1 Division of Small Animal Internal Medicine, Department of Clinical Veterinary Medicine, Vetsuisse Faculty, University of Bern, Bern, Switzerland; 2 Clinical Laboratory, Vetsuisse Faculty, University of Zurich, Zurich, Switzerland; 3 Center for Clinical Studies, Vetsuisse Faculty, University of Zurich, Zurich, Switzerland; 4 Clinic of Reproductive Medicine, Vetsuisse Faculty, University of Zurich, Zurich, Switzerland; 5 Clinical Diagnostic Laboratory, Department of Clinical Veterinary Medicine, Vetsuisse Faculty, University of Bern, Bern, Switzerland; 6 Clinic for Small Animal Internal Medicine, Vetsuisse Faculty, University of Zurich, Zurich, Switzerland

**Keywords:** Cat, *Cytauxzoon*, Piroplasm, Protozoan, Theileridae, Tick-borne disease, Transfusion-transmitted diseases, Vector-borne disease, PCR

## Abstract

**Background:**

Cytauxzoonosis is an emerging tick-borne disease of domestic and wild felids. *Cytauxzoon felis* induces severe and often fatal disease in domestic cats. In Europe, clinical and subclinical infections caused by *Cytauxzoon* sp. are described. We report the first cases of *Cytauxzoon* sp. infection in domestic cats in Switzerland.

**Methods:**

Clinical and laboratory data and results of PCR analyses were collected from *Cytauxzoon* sp. PCR-positive cats and the cats followed for up to 851 days.

**Results:**

The cases were three two-month old kittens from the same litter (Cases 1–3) and two adult domestic shorthair cats (Cases 4 and 5). The cats originated from the north-west and west of Switzerland. Cases 1–3 presented with moderate to severe regenerative anaemia and intraerythrocytic inclusions. *Cytauxzoon* sp*.* was confirmed by PCR and sequencing*.* The kittens made a clinical and haematological recovery after blood transfusion and/or treatment with azithromycin and atovaquone, but erythroparasitaemia persisted. Case 4 presented with severe non-regenerative anaemia. Case 5 was healthy and used as a blood donor for Case 4. Following blood transfusion, Case 4 showed intraerythrocytic inclusions, and *Cytauxzoon* sp*.* was confirmed in both Cases 4 and 5 using PCR and sequencing. Case 4 achieved clinical and haematological remission after treatment with azithromycin, atovaquone and immunosuppressive drugs. Eight months later, Case 4 was presented again with anaemia but tested *Cytauxzoon* sp. PCR-negative. Sequencing of 1637 bp of the *18S* rRNA gene of *Cytauxzoon* sp*.* revealed 100% nucleotide sequence identity among isolates of Cases 1–3 and between isolates of Cases 4 and 5, and 99% sequence identity between isolates of all cases. Phylogenetic analysis revealed the closest relationship of the Swiss isolates to *Cytauxzoon* sp*.* isolates from domestic cats and wild felids from France, Spain and Romania and to *Cytauxzoon manul* from a Pallas’s cat.

**Conclusions:**

This is the first report of *Cytauxzoon* sp*.* infection in domestic cats in Switzerland. It is also the first report of infection in very young kittens and transmission of *Cytauxzoon* sp*.* to an adult cat by transfusion of blood from an asymptomatic cat. The cats recovered but some developed chronic asymptomatic erythroparasitaemia for up to 28 months. Domestic cats may act as reservoirs for *Cytauxzoon* sp. in Europe and blood donor cats should be screened for this agent by PCR.

## Background

Cytauxzoonosis is a tick-borne disease caused by the apicomplexan haemoparasites *Cytauxzoon* spp. The best-characterized species, with several genotypes, is *Cytauxzoon felis* [1, 2]. Both *Dermacentor variabilis* and *Amblyomma americanum* ticks have been shown to transmit *C. felis* in experimental settings [3, 4].

In domestic cats, *C. felis* infection is generally rapidly progressive and associated with high mortality. Affected cats show fever, depression, anorexia, vomiting, pale mucous membranes, icterus and hepatosplenomegaly. Clinicopathological signs include non-regenerative anaemia, leukopenia, thrombocytopenia and hyperbilirubinaemia [5]. The schizogonous phase of the organism is associated with the development of severe clinical disease in domestic cats. Schizont-laden macrophages obstruct small vessels in numerous tissues and lead to circulatory impairment and multi-organ failure [6]. Despite high mortality associated with *C. felis* infection in domestic cats, both survival after clinical infection and subclinical persistent parasitaemia have recently been documented [7–9]. This may suggest a shift in parasite vector-host interactions, although the reason for this is unclear [8, 9]. Chronically infected cats may harbour the agent for prolonged periods, thereby acting as reservoirs of *C. felis* [9, 10]. However, the main natural reservoir of *C. felis* in the USA is thought to be the wild bobcat *(Lynx rufus)*. Infections in this species are most often subclinical [4, 11, 12], although fatal cytauxzoonosis has been documented in bobcats, tigers and lions [12–16]. Factors affecting pathogenicity of *C. felis* in wild felids are largely unknown. However, the schizogonous phase in bobcats appears to be short and is rarely associated with clinical signs [12].

Most reports of cytauxzoonosis are of domestic cats in the south-eastern, central and mid-Atlantic states of the USA. Isolates closely related to *C. felis* have also been documented in domestic and wild felids in Brazil [17–21]. More recently, infection with other species of *Cytauxzoon* spp. in wild and domestic cats has been described. Species identified in the Eastern Hemisphere include *Cytauxzoon manul* in a Pallas’s cat (*Felis manul*, syn. *Otocolobus manul*) [22, 23] and *Cytauxzoon* sp. in domestic cats, Eurasian lynx (*Lynx lynx*) and European wildcats (*Felis silvestris*) [24–36]. These isolates were genetically different from *C. felis* and were most closely related to *C. manul.* Infections with *Cytauxzoon*-like organisms have also been described in domestic and wild felids in Iran, Germany and Zimbabwe [16, 37–39], and most recently in meerkats (*Suricata suricatta*) in South Africa [40].

*Cytauxzoon* sp. is generally considered less virulent in domestic cats than *C. felis*, but the clinical significance of *Cytauxzoon* sp. infection in domestic cats in Europe remains unclear. One study found a prevalence of up to 22.9% in cats in northeastern Italy [24], the majority of which appeared clinically healthy. Indeed, no association between infection and clinical status, laboratory findings or mortality was found, and only 7% were anaemic at the time of diagnosis. Conversely, symptomatic and even fatal infections have also been documented [24, 30, 31, 41]. These cases presented with a variety of clinical signs, including lethargy, anorexia, weight loss, pyrexia, pale mucous membranes, diarrhoea, vomiting and pleural or peritoneal effusion. In addition, some cats exhibited clinical signs not typically associated with piroplasm infections, including stomatitis, ulcerative dermatitis, circling and vocalizations [24, 31]. However, concomitant diseases, such as neoplasia, intracranial disorders or feline infectious peritonitis that may cause similar clinical signs were not always excluded [24, 30].

Here we report the first clinical cases of *Cytauxzoon* sp*.* infection in domestic cats in Switzerland and the molecular characterization of the isolates. The cases are three two-month old kittens with symptomatic natural infection, and an adult cat with suspected blood transfusion-transmitted infection as well as the asymptomatic blood donor of this cat.

## Methods

### Inclusion criteria, sample and data collection

Cats were included if they tested PCR-positive for *Cytauxzoon* spp. and if the PCR products were sequenced and showed > 95% sequence identity with *Cytauxzoon* spp. The cases were presented to the Section of Small Animal Reproduction, Vetsuisse Faculty, University of Zurich (Cases 1–3), and to the Small Animal Clinic of the Vetsuisse Faculty, University of Bern (Cases 4 and 5). Signalment, medical history, clinical examination findings, treatment and results of haematology, biochemistry, blood smear examination and PCR analyses were collected (Tables 1, 2, 3 and 4). Four cats were followed for 851 (Cases 1–3) and 264 (Case 4) days, respectively.

**Table 1:** Patient characteristics of the five cats infected with *Cytauxzoon* sp

Case number	Date of presentation	Geographical location	Outdoor access	No. of other cats in household	Breed	Gender	Age (months)	Anamnesis	Clinical examination findings
1	21.07.14	Montignez	yes	2	DSH	f	2	Inappetence, lethargy	Pallor, tachycardia, tachypnoea
2	22.07.14	Montignez	yes	2	DSH	m	2	No clinical signs	Pallor, tachycardia
3	22.07.14	Montignez	yes	2	DSH	f	2	No clinical signs	Pallor, tachycardia
4	14.11.15	Vallorbe	no ^a^	2	DSH	f	13	Anorexia, lethargy, weight loss	Pallor, lethargy, underweight, tachycardia, tachypnoea, heart murmur
5	14.11.15	Vallorbe	yes	0	DSH	mc	28	No clinical signs	Unremarkable

*Abbreviations*: *DSH* domestic shorthair, *f* female intact, *m* male intact, *mc* male castrated

^a^not since three-months of age

**Table 2:** Haematological analyses in the five cats infected with *Cytauxzoon* sp. Results outside the reference interval are shown in bold font. Cases 1–3 were treated with azithromycin and atovaquone from day 1 to 10 and from day 77 to 86. Case 4 was treated with azithromycin from day 6 to 21 and from day 260 to 270 and with atovaquone from day 11 to 21

Parameter	Case	Day 0 (presentation)	Week 1 (days 1–7)	Week 2 (days 8–14)	Weeks 4–5 (days 22–35)	Week 10 (days 64–70)	Week 17 (days 113–119)	Week 38 (days 260–266)	Week 122 (days 848–854)
PCV or haematocrit (RI) (%)	1	**8**^**a**^ (33–34)^c^	**22**^a^ (33–34)^c^	**30**^b^ (33–34)^c^	**29**^b^ (32–35)^c^	34^b^ (34–36)^c^	**40**^b^ (30–37)^c^	na	37^b^ (33–45)
	2	**18**^a^ (33–34)^c^	**23**^a^ (33–34)^c^	33^b^ (33–34)^c^	**29**^b^ (32–35)^c^	34^b^ (34–36)^c^	**38**^b^ (30–37)^c^	na	36^b^ (33–45)
	3	**14**^a^ (33–34)^c^	**19**^a^ (33–34)^c^	**25**^a^ (33–34)^c^	**28**^b^ (32–35)^c^	**29**^b^ (34–36)^c^	36^b^ (30–37)^c^	na	**32**^b^ (33–45)
	4	**8**^**a**^ (27–47)	**9**^b^ (27–47)	**20**^b^ (27–47)	27^b^ (27–47)	na	na	**13**^b^ (27–47)	na
	5	35^**b**^ (27-47)	na	na	41^b^ (27–47)	na	na	na	na
MCV (RI) (fl)	1	**71 (**47–49)^c^	na	**41** (47–49)^c^	na	**34** (42–45)^c^	na	na	**37** (41–49)
	2	na	na	51 (47–49)^c^	na	**38** (42–45)^c^	na	na	**40** (41–49)
	3	na	na	47 (47–49)^c^	na	**36** (42–45)^c^	na	na	41 (41–49)
	4	na	**36** (37–55)	**61** (37–55)	47 (37–55)	na	na	54 (37–55)	na
	5	na	na	na	41 (37–55)	na	na	na	na
MCHC (RI) (g/l)	1	**330** (291–299)^c^	na	**350** (291–299)^c^	na	**360** (308–324)^c^	na	na	350 (330–360)
	2	na	na	**320** (291–299)^c^	na	**350** (308–324)^c^	na	na	350 (330–360)
	3	na	na	**290** (291–299)^c^	na	**360** (308–324)^c^	na	na	360 (330–360)
	4	na	**392** (263–359)	317 (263–359)	336 (263–359)	na	na	351 (263–359)	na
	5	na	na	na	324 (263–359)	na	na	na	na
Reticulocytes (RI) (×10^9^/l)	1	**250** (≤ 60)^c^	na	na	na	na	na	na	na
	2	**570** (≤ 60)^c^	na	na	na	na	na	na	na
	3	**470** (≤ 60)^c^	na	na	na	na	na	na	na
	4	na	13 (4–94)	**185** (4–94)	19 (4–94)	na	na	**163** (4–94)	na
	5	na	na	na	20 (4–94)	na	na	na	na
Leucocytes (RI) (×10^9^/l)	1	**32** (22–26)^c^	na	**12** (22–26)^c^	na	**10** (19–21)^c^	na	na	9 (5–13)
	2	na	na	**14** (22–26)^c^	na	**10** (19–21)^c^	na	na	8 (5–13)
	3	na	na	**12** (22–26)^c^	na	**8** (19–21)^c^	na	na	8 (5–13)
	4	na	7 (7–15)	10 (7–15)	11 (7–15)	na	na	8 (7–15)	na
	5	na	na	na	10 (7–15)	na	na	na	na
Platelets (RI) (×10^9^/l)	4	na	**86** (180–430)	clumps	407 (180–430)	na	na	clumps	na
	5	na	na	na	clumps	na	na	na	na

*Abbreviations*: *na* not applicable, *RI* reference interval, *PCV* packed cell volume, *MCV* mean corpuscular volume, *MCHC* mean corpuscular haemoglobin concentration

^a^Values based on PCV

^b^Values based on automated haematocrit

^c^Reference intervals for kittens based on [44]; reference intervals change with the age of the kittens

**Table 3:** Biochemistry analyses in the five cats infected with *Cytauxzoon* sp. Results outside the reference interval are shown in bold font

Case	Time point	Bilirubin (RI) (μmol/l)	Urea (RI) (mmol/l)	Creatinine (RI) (μmol/l)	Total protein (RI) (g/l)	Albumin (RI) (g/l)	AP (RI) (IU)	ALT (RI) (IU)	Sodium (RI) (mmol/l)	Potassium (RI) (mmol/l)	Phosphorus (RI) (mmol/l)
1	Day 14	0.4 (–1.7)^a^	9.4 (5.7–11.8)^a^	39 (14–111)^a^	56 (48–65)^a^	29 (24–30)^a^	129 (≤ 564)^a^	**63** (10–50)^a^	152 (143–160)^a^	4.3 (3.7–6.1)^a^	2.7 (2.1–3.3)^a^
2	Day 14	0.2 (–1.7)^a^	7.2 (5.7–11.8)^a^	37 (14–111)^a^	56 (48–65)^a^	26 (24–30)^a^	97 (≤ 564)^a^	32 (10–50)^a^	150 (143–160)^a^	4.3 (3.7–6.1)^a^	2.6 (2.1–3.3)^a^
3	Day 14	0.8 (–1.7)^a^	8.8 (5.7–11.8)^a^	37 (14–111)^a^	61(48–65)^a^	28 (24–30)^a^	144 (≤ 564)^a^	**62** (10–50)^a^	151 (143–160)^a^	4.3 (3.7–6.1)^a^	2.6 (2.1–3.3)^a^
4	Day 2	1.1 (0–6.2)	10.8 (6.5–12.2)	69 (52–138)	75 (55–76)	31 (30–41)	8 (0–93)	77 (12–77)	148 (144–159)	**2.8** (3.1–4.9)	1.1 (0.8–1.9)
5	Day 0	na	na	94 (71–212)	na	na	na	na	157 (151–157)^b^	4.2 (3.1–4.6)^b^	na

*Abbreviations*: *na* not applicable, *RI* reference interval, *AP* alkaline phosphatase, *ALT* alanine aminotransferase

^a^Reference intervals for kittens based on [44, 45]

^b^Reference intervals for Rapidpoint 400 analyses based on [43]

**Table 4:** Identification of intraerythrocytic inclusions and PCR results in the five cats infected with *Cytauxzoon* sp

Parameter	Case	Day 0 (presentation)	Week 1 (days 1–7)	Week 2 (days 8–14)	Weeks 4–5 (days 22–35)	Week 10 (days 64–70)	Week 17 (days 113–119)	Weeks 38–39 (days 260–273)	Week 122 (days 848–854)
Intraerythrocytic inclusions	1	positive	positive	positive	na	positive	positive	negative	na
	2	positive	positive	positive	na	positive	negative	negative	na
	3	positive	positive	positive	na	positive	negative	negative	na
	4	negative	positive	positive	negative	na	na	negative	na
	5	na	na	na	na	na	na	na	na
PCR for *Cytauxzoon* sp.^a^	1	na	positive	na	positive	positive	positive	positive	positive
	2	positive	positive	na	positive	positive	positive	positive	positive
	3	positive	positive	na	positive	positive	positive	positive	positive
	4	negative	positive	na	positive	na	na	negative	na
	5	na	na	na	positive	na	na	na	na

*Note*: Cases 1–3 were treated with azithromycin and atovaquone from day 1 to 10 and from day 77 to 86. Case 4 was treated with azithromycin from day 6 to 21 and from day 260 to 270 and with atovaquone from day 11 to 21

*Abbreviation*: *na* not applicable

^a^Screened by real-time PCR and confirmed by conventional PCR and sequencing

### Haematology and blood biochemistry

Haematology and blood biochemistry were performed at the Clinical Laboratory, Vetsuisse Faculty, University of Zurich (Cases 1–3), using a Sysmex XT-2000iV (Sysmex Corporation, Kobe, Japan) [42] and a Cobas Integra 800 instrument (Roche Diagnostics AG, Rotkreuz, Switzerland), and at the Clinical Diagnostic Laboratory, Vetsuisse Faculty, University of Bern (Cases 4 and 5), using an Advia 2120 (Siemens Healthcare AG, Zurich, Switzerland) and a Cobas c501 (Roche Diagnostics AG), respectively. Haematocrit and blood biochemistry in Case 5 at first presentation was measured on a Rapidpoint 400 instrument (Siemens Healthcare Diagnostics GmbH, Zurich, Switzerland) and on an IDEXX Vettest Chemistry Analzyer (IDEXX Laboratories, Inc., Westbrook, ME, USA). Modified Wright-Giemsa-stained blood smears (AMES Hema Tek slide stainer, Bayer, Zurich, Switzerland or Hema-Tek 2000, Siemens) were made from fresh EDTA-anticoagulated blood. The Coombs’ test was performed using the microdilution plate method at the Clinical Diagnostic Laboratory, Vetsuisse Faculty, University of Bern using a commercial feline Coombs’ reagent (ImmunO, MP Biomedicals Llc., Solon, OH, USA). The laboratories’ own and published reference intervals [43] were used for adult cats and published reference intervals were used for kittens [44, 45].

### Nucleic acid extraction

Total nucleic acid (TNA) extraction was performed from 100 μl of EDTA whole blood with the MagNa Pure LC (Roche Diagnostics AG) using the MagNa Pure LC TNA Isolation Kit (Roche Diagnostics AG). A negative control consisting of 100 μl phosphate-buffered saline was concurrently prepared with each batch of extractions to monitor for cross-contamination. Extracted nucleic acids were stored at -20 °C prior to PCR analyses.

### Diagnostic assays

Detection of feline leukaemia virus (FeLV) p27 antigen and feline immunodeficiency virus (FIV) antibody was performed using commercial lateral-flow ELISA tests (SNAP® Kombi Plus FIV/FeLV, IDEXX Diavet AG, Bäch, Switzerland). Detection of FeLV provirus, *Mycoplasma haemofelis*, “*Candidatus* Mycoplasma haemominutum” and “*Candidatus* Mycoplasma turicensis” was performed using EDTA-anticoagulated blood and species-specific TaqMan real-time PCR assays [46–48]. For the detection of *Cytauxzoon* sp. in EDTA-anticoagulated blood, a TaqMan real-time PCR assay that amplifies 69 bp of the *18S* rRNA gene was used. The assay is based on the following primers and probe: forward primer Cytsp. 1525f (5'-GAA TGC CTA GTA GAC GCG AGT CA-3'), reverse primer Cytsp. 1593r (5'-ACG GGC GGT GTG TAC AAA G-3'), and probe Cytsp. 1549p (5'-FAM- CAG CTC GTG TCG ATT ACG TCC CTG C-TAMRA-3'). The primers and probe were designed to specifically detect the *18S* rRNA sequences of *Cytauxzoon* spp. present in the NCBI GenBank. The PCR mixture comprised 12.5 μl of 2× qPCR^TM^ Mastermix (Eurogentec, Seraing, Belgium), 1.125 μl of each primer and 0.625 μl of the probe, and 5 μl TNA made up to 25 μl with nuclease-free water. Cycling conditions were as follows: 2 min at 50 °C and 10 min at 95 °C, followed by 45 cycles of 15 s at 95 °C and 1 min at 60 °C. Cycle threshold (Ct) values were used to estimate the *Cytauxzoon* sp. burden in the sample. All real-time PCRs were run on an ABI 7500 Fast Real-Time PCR system (Applied Biosystems, Rotkreuz, Switzerland). Positive and negative controls that were run with each assay consisted of DNA from a *Cytauxzoon* sp. PCR-positive Iberian lynx (confirmed by sequencing) and of nuclease-free water, respectively. *Cytauxzoon* sp. PCR-positive and follow-up samples were further analysed with a conventional PCR assay that amplifies 221 bp of the *18S* rRNA gene of *Cytauxzoon* spp. [35]. Briefly, the assay is based on the following primers: forward primer Cytfelis.203f (5'-AGA CCY YAA ACC ATC CCG CT-3') and reverse primer Cytfelis.423r (5'-CCT GCT GCC TTC CTT AGA TG-3'). The PCR mixture comprised 2.5 units of Taq DNA Polymerase (Sigma-Aldrich, Buchs, Switzerland), a final concentration of 500 nM of each primer, 200 μM dNTPs (Sigma-Aldrich), 2.5 μl of 10× PCR Buffer (Sigma-Aldrich), and 2.5 μl of template made up to a final volume of 25 μl with nuclease-free water. The thermal cycling conditions were 95 °C for 5 min, 35 cycles at 95 °C for 30 s, 70 °C for 45 s, and 72 °C for 1 min, and 72 °C for 10 min. The PCR was run on the Biometra Tpersonal thermal cycler (Biometra, Göttingen, Germany). The PCR products were separated on a 2% agarose gel and bands of appropriate size (221 bp) were initially sequenced at a commercial laboratory (Microsynth AG, Balgach, Switzerland) using the amplification primers.

### *18S* rRNA amplification and sequencing

The near complete *Cytauxzoon* sp. *18S* rRNA gene sequence (1637 bp) was determined for Case 1 on days 7 and 28, for Cases 2 and 3 on days 0 and 28, for Case 4 on day 5, and for Case 5 on day 24 using published methods [49]. Briefly, the *18S* rRNA gene was amplified using the forward primer Cytlblynx.23f (5'-GCC ATG CAT GTC TAA GTA TAA GC-3') and the reverse primer Cytlblynx.1659r (5'-CGC GCC TAA CGA ATT AGA AG-3'). The PCR reaction comprised 0.5 μl of Phusion Hot Start II DNA Polymerase (Thermo Fisher Scientific, Reinach, Switzerland), 5 μl of HF Phusion Buffer (Thermo Fisher Scientific), a final concentration of 0.5 μM of each primer, 0.2 mM dNTPs (Sigma-Aldrich), and 2.5 μl of template made up to a final volume of 25 μl with nuclease-free water. The thermal cycling conditions were 98 °C for 3 min, 35 cycles at 98 °C for 10 s, 56 °C for 30 s, and 72 °C for 1 min, and 72 °C for 10 min. PCR products were separated on a 1.5% agarose gel and bands of appropriate size (1,637 bp) were cut, extracted using the MinElute® gel extraction kit (Qiagen GmbH, Hilden, Germany) and the PCR products were sequenced. Nucleotide sequences have been submitted to GenBank under accession numbers MF503141–MF503148.

### Genetic and phylogenetic analyses

Sequences were edited and assembled using Geneious® v.9.1.7 software (Biomatters Limited, Auckland, New Zealand) [50]. Phylogenetic analysis was conducted using MEGA7 [51]. Sequences were aligned to additional reference sequences retrieved from GenBank using the Clustal W algorithm. The phylogenetic tree was inferred using the maximum-likelihood method using a distance matrix corrected for nucleotide substitutions based on the Kimura 2-parameter model [52]. Initial tree(s) for the heuristic search were obtained automatically by applying neighbour-joining and BioNJ algorithms to a matrix of pairwise distances estimated using the maximum composite likelihood approach, and then selecting the topology with superior log likelihood value. Codon positions included were 1st+2nd+3rd+noncoding. All positions containing gaps and missing data were eliminated. The dataset was resampled 1000 times to generate bootstrap values [53].

## Results

### Patient characteristics and geographical location

**Fig. 1:**
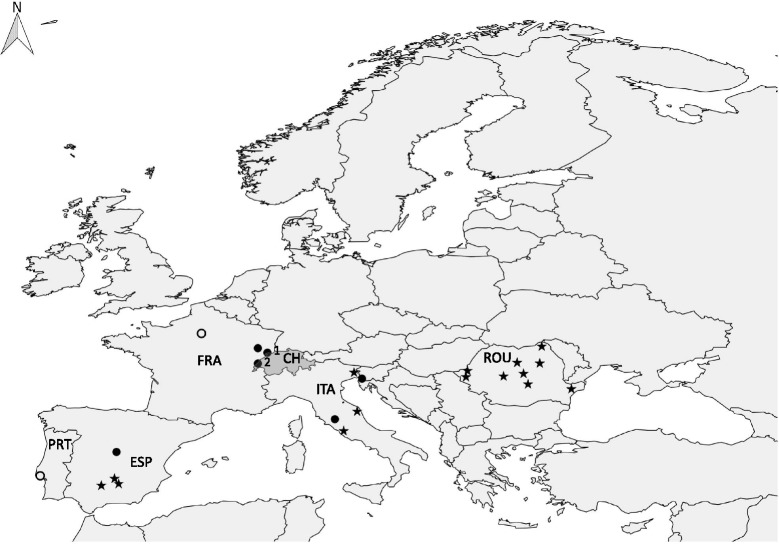
Map of Europe indicating the geographical location of Cases 1–5 and published cases. The geographical location of the present and published *Cytauxzoon* sp. cases in domestic cats and wild felids in Europe is indicated with dots and stars, respectively [24–36]. Numbers 1 and 2 indicate the locations of Cases 1–3 (Montignez, Switzerland) and Cases 4–5 (Vallorbe, Switzerland), respectively (see also Table 1). Two cases in domestic cats for which the location within the country was not specified in the publication [30, 31] were allocated to the capital city of the country (indicated as open dots). *Abbreviations*: PRT, Portugal; ESP, Spain; FRA, France; CH, Switzerland; ITA, Italy; ROU, Romania

Details of patient characteristics and geographical locations of the five cats are shown in Table 1 and Fig. 1. All cases originated from the north-west or west of Switzerland and Cases 1–4 lived in multi-cat households. Cases 1–3 were unvaccinated two-month old siblings that lived in the same household and had been adopted by an animal welfare organization at five-weeks of age. At the time of adoption, Cases 1–3 had severe tick infestation. No data were available from the queen of these cats. Cases 4 and 5 were adult cats originating from the same village but living in separate households. Case 4 was not vaccinated or given preventive medication against fleas or ticks and lived with two other cats that were clinically unremarkable. Case 5 was the blood donor for Case 4 and was a clinically healthy cat with no previous medical history. Case 5 had not been previously used as a blood donor and was regularly vaccinated and given preventive treatment against endo- and ectoparasites.

### Anamnesis and clinical examination findings of Cases 1–5

Case 1 was presented because of lethargy, pallor and reduced appetite. No signs of illness had been noted in the siblings, Cases 2 and 3. On clinical examination, all kittens were tachycardic and had pale mucous membranes. Case 4 was presented with anorexia, lethargy and weight loss. The cat had been referred by the private veterinarian because of severe anaemia and had been treated once with hydrocortisone and vitamin K. Upon clinical examination, Case 4 was underweight and severely lethargic. Marked pallor, tachycardia, tachypnoea, and a systolic heart murmur were noted. Case 5 was asymptomatic and clinical examination was unremarkable.

### Haematology and biochemistry

**Fig. 2:**
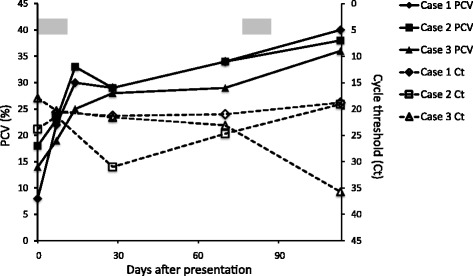
Course of PCV and *Cytauxzoon* sp. parasitaemia in Cases 1–3. The PCV (solid lines) and *Cytauxzoon* sp. parasitaemia (dashed lines) during 133 days following presentation are shown. The time-span during which the cats received medical therapy with atovaquone and azithromycin (days 1–10 and days 77–86) is indicated with a grey box. The *Cytauxzoon* sp. parasitaemia is represented by the cycle threshold (Ct) value of the real-time PCR. The Ct value is inversely proportional to the relative amount of target nucleic acid in the real-time PCR reaction

Results of haematological and biochemical analyses in the five cases are detailed in Tables 2 and 3. At presentation, Cases 1–3 had moderate to severe regenerative anaemia, and Case 1 had mild leukocytosis. By week 2 (4 days after a 10-day course of therapy), haematology revealed only mild anaemia (Fig. 2, Table 2). At week 17 (4 weeks after the end of a second 10-day course of therapy) the haematocrit was normal in all three cases. Mildly elevated alanine aminotransferase values (Cases 1 and 3) were evident at week 2.

Case 4 was presented with severe anaemia and received a whole blood transfusion from Case 5 at admission. The anaemia was non-regenerative in week 1 and became strongly regenerative in week 2 (Table 2). Both the haematocrit and reticulocyte counts normalized in week 4, followed by relapse with severe anaemia in week 38. The Coombs’ test was positive (up to a dilution of 1:128) at week 1 and negative in weeks 4 and 38. Besides mild hypokalaemia, no abnormalities were present in blood biochemistry in Case 4. Case 5 had normal haematocrit and urea and electrolyte levels at the time of blood donation and no abnormalities on haematological examination performed at week 4.

### Diagnostic imaging and cytology

Diagnostic imaging and cytology results were available for Case 4. Thoracic radiographs at admission were unremarkable. Abdominal ultrasound revealed evidence of pancreatitis and enteritis in week 1, and mild splenomegaly in week 38. Cytological examination of splenic fine needle aspirates in week 38 revealed extramedullary haematopoiesis.

### Blood smear examinations

**Fig. 3:**
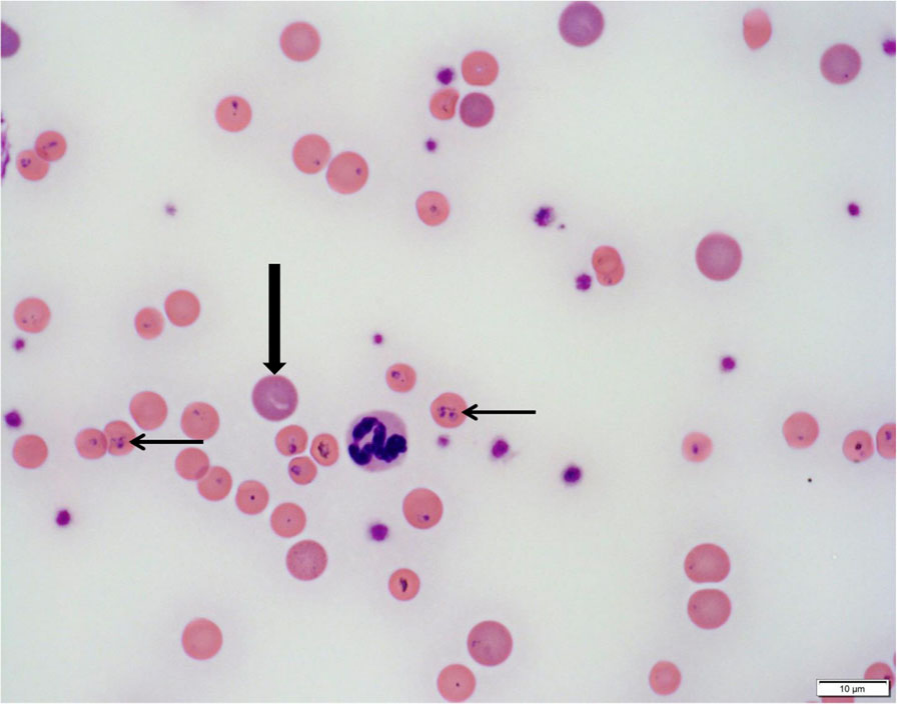
Modified Wright-stained blood smear of Case 1. Intraerythrocytic inclusions with characteristic signet ring-shaped piroplasms consistent with *Cytauxzoon* sp. are present in most erythrocytes (examples indicated by thin arrows). Polychromatophilic red blood cells, supporting regenerative anaemia, are present (example indicated by a thick arrow). 1000× magnification. *Scale-bar*: 10 μm

Cases 1–3 showed intraerythrocytic inclusions consistent with *Cytauxzoon* spp. at first admission and in the follow-up examinations up to weeks 10–17 (Table 4). These were characterized by a signet-ring shape with a round thickening of nuclear material at one point of the ring (Fig. 3). Intraerythrocytic inclusions, consistent with *Cytauxzoon* spp. were absent in Case 4 at first admission, but were detected in weeks 1 and 2, five and eleven days after receiving a blood transfusion from Case 5, respectively (Table 4). In weeks 4 and 38, intraerythrocytic inclusions were not detected.

### *Cytauxzoon* sp. PCR

Cases 1–3 tested *Cytauxzoon* sp. PCR-positive in every blood sample collected from admission until the end of the 851-day follow-up period (Table 4). Case 4 tested PCR-negative for *Cytauxzoon* sp. at admission, but was *Cytauxzoon* sp. PCR-positive five days after receiving a blood transfusion from Case 5. The cat remained PCR-positive in week 4, albeit with a marked reduction in parasitaemia, and was PCR-negative at week 38. Blood from Case 5 collected five weeks after blood donation tested PCR-positive for *Cytauxzoon* sp. No follow-up was available for this cat.

### Results for FeLV, FIV and feline haemoplasmas

Cases 1–5 tested PCR-negative for *Mycoplasma haemofelis*, “*Candidatus* Mycoplasma haemominutum” and “*Candidatus* Mycoplasma turicensis”, and negative for FIV and FeLV (p27, Cases 1–4; provirus, Cases 4–5).

### Treatment, clinical course and outcome

Cases 1–3 were treated twice with atovaquone (15 mg/kg PO q8h) and azithromycin (10 mg/kg PO q24h) for 10 days (weeks 1–2 and 11–12, respectively). Case 1 also received a 24 ml whole blood transfusion from a healthy donor cat. In week 2, the cats were clinically unremarkable and remained so throughout the 851-day follow-up period.

At admission, Case 4 received a 50 ml whole blood transfusion from Case 5. Treatment with azithromycin (10 mg/kg PO q24h for 26 days) and atovaquone (15 mg/kg PO q8h for 11 days) was initiated in weeks 1 and 2, respectively. The cat was also given immunosuppressive treatment (prednisolone, 2 mg/kg/d and cyclosporine, 5 mg/kg/d, in tapering doses over two months), antibiotics (doxycycline, 10 mg/kg/d for 10 days), and gastroprotective and antiemetic medication. The cat’s general condition was slightly improved in week 1, but intermittent fever was noted. In week 2, the cat was normothermic with a good appetite. In week 4, the cat was clinically unremarkable but was presented again in week 38 with lethargy, fever, pallor, tachycardia and severe regenerative anaemia (Table 2). The cat was treated with azithromycin and immunosuppressive doses of prednisolone. The cat rapidly improved and was discharged after five days of hospitalization. Case 4 was then lost for further follow-up. No treatment was given to Case 5 and no follow-up data was available from this cat.

### *Cytauxzoon* sp. sequencing

**Fig. 4:**
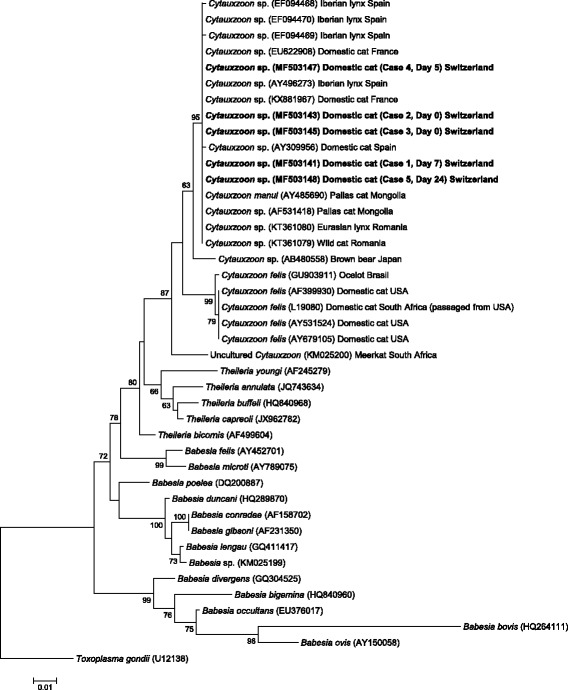
Molecular phylogenetic analysis by maximum likelihood method of the *18S* rRNA gene. The tree with the highest log likelihood (-3233.55) is shown. The percentage of trees in which the associated taxa clustered together is shown next to the branches. The tree is drawn to scale, with branch lengths measured in the number of substitutions per site. The analysis involved 42 nucleotide sequences (GenBank accession numbers are indicated). There were a total of 726 positions in the final dataset

The *Cytauxzoon* sp. *18S* rRNA gene sequences from Cases 1, 2 and 3 showed 100% sequence identity at all time points. The *Cytauxzoon* sp. *18S* rRNA gene sequences from Cases 4 and 5 also showed 100% sequence identity. Sequence comparison between isolates from Cases 1–3 and Cases 4 and 5 showed a single nucleotide mismatch, resulting in a sequence identity of 99%. The isolates of Cases 1–5 showed highest sequence identity with *Cytauxzoon* sp. isolates from domestic cats from France (GenBank: EU622908, 99–100% sequence identity) and Spain (GenBank: AY309956, 99% sequence identity), and with *C. manul* from Mongolia (GenBank: AY485690, 99% sequence identity). Sequence comparison revealed only 96% sequence identity with a *C. felis* isolate from the USA (GenBank: AY679105). Phylogenetic analysis showed that the Swiss isolates clustered together with European *Cytauxzoon* sp. isolates from domestic and wild felids, and with *C. manul* from a Pallas’s cat (Fig. 4).

## Discussion

Since the first report in the mid-1970s [54], feline cytauxzoonosis caused by *C. felis* has been considered an emerging disease with an expanding case distribution from the south-central to the south-east and mid-Atlantic regions of the USA [55]. In recent years, infections with molecularly distinct *Cytauxzoon* spp. have been described in domestic cats in Europe, i.e. in Italy, France, Spain and Portugal [24, 29–31, 36, 41]. The present report is the first description of cases of *Cytauxzoon* sp. infection in domestic cats in Switzerland. In agreement with previous European studies, the agent detected in the five cases was phylogenetically distinct from *C. felis* and most closely related to *C. manul* [22, 23]. Our results further support the hypothesis that different *Cytauxzoon* species or strains exist in different geographical locations and that the species might differ in pathogenicity. To further resolve *Cytauxzoon* taxonomy, phylogenetic studies based on additional genes (mitochondrial genes or whole genome sequencing) should be performed.

Given that infection of domestic cats with *C. felis* occurs in areas with both a natural reservoir host (the American bobcat) and a tick vector (*Amblyomma americanum* or *Dermacentor variabilis*) [56], similar conditions are likely to exist for *Cytauxzoon* sp. infections in Europe. Although reservoirs in Europe have not been definitively identified, infections in European wildcats in Italy and Romania [33, 34], in the Iberian lynx in Spain [28] and in the Eurasian lynx in Romania and Switzerland ([34], M.L. Meli, personal communication) are documented. The domestic cats described in the present report originated from two villages in the north-west or west of Switzerland. Both villages are located near the French border and 70–140 km away from Saint Sauveur (Bourgogne-Franche-Comté region) where a symptomatic *Cytauxzoon* sp. infection occurred in a domestic cat in France [31]. The north-west and west of Switzerland host a stable population of the Eurasian lynx, reintroduced in Switzerland in the 1970s, and of the European wildcat [57, 58]. Similar to bobcats serving as the main reservoir of *C. felis* in the USA [6], it is likely that the Eurasian lynx plays a central role as a reservoir of *Cytauxzoon* sp. in Switzerland. The role of the European wildcat as a reservoir of *Cytauxzoon* sp. is not clear, but given that they might live in close proximity and even interbreed with domestic cats [59], a transmission of *Cytauxzoon* sp. between domestic and wild cats *via* ticks seems possible. The tick vector for *Cytauxzoon* sp. in Europe is not known. Ticks of the species *Ixodes* spp.*, Dermacentor* spp. and *Rhipicephalus* spp. have all been described in Switzerland [60–62] and could be involved in transmission. All cases in this study lived in a rural area. An association between *Cytauxzoon* sp. infection and living in rural areas and outdoor access was recently documented in domestic cats in Spain and Italy, respectively [24, 36].

Cases 1–3 were siblings from the same litter and were presented at only eight weeks of age. *Cytauxzoon* sp. infection was also documented in two six- to seven-month-old siblings in Italy [41]. Experimental studies failed to demonstrate perinatal transmission of *C. felis* in domestic cats [63]. Since all kittens described in the present report had severe tick infestation at the time of adoption, a vector-borne transmission of *Cytauxzoon* sp. infection is likely. Unfortunately, clinical data and blood samples were not available from the queen to exclude vertical transmission in these cases.

The pathogenesis of *Cytauxzoon* sp. infection in domestic cats is not fully elucidated but appears to differ from that caused by *C. felis*. The latter is generally associated with high mortality [64], although asymptomatic infections have been described in recent years [7–10]. The acute stage of disease is caused by schizonts, large structures within mononuclear cells that occlude blood vessels in various organs and result in circulatory impairment and multiorgan dysfunction. Although the schizogonous phase has not yet been identified in *Cytauxzoon* sp. infected cats [24, 31, 41], it is very likely that *Cytauxzoon* sp. also undergoes schizogony given the molecular phylogeny of *Cytauxzoon* spp. organisms [65]. In bobcats, the natural reservoir of *C. felis*, schizogony does occur but appears to be limited in duration and rarely causes clinical disease [12].

Both symptomatic and asymptomatic *Cytauxzoon* sp. infections have been documented in previous and the present reports [24, 30, 31, 41]. Besides the blood donor (Case 5), all cats in the present report showed clinical signs that could be attributed to *Cytauxzoon* sp. infection. As erythroparasitaemia was only observed after blood transfusion in Case 4, the extent to which infection with *Cytauxzoon* sp. contributed to clinical disease in this cat is unclear. Given the initial negative results for infectious aetiologies, including *Cytauxzoon* spp., immune-mediated disease was suspected to be the cause of initial clinical presentation. The positive Coombs’ test in Case 4 may reflect a primary immune-mediated disorder but may also have been due to previous blood transfusion. No retroviral or haemoplasma infections were present in the cats that could have exacerbated clinical disease. However, Cases 1–4 may have been immunocompromised due to young age or immunosuppressive therapy, which may have predisposed them to the development of clinical disease. Similar to previous reports [24, 30, 31, 41], the cases described herein presented with nonspecific clinical signs, such as anorexia and lethargy. On physical examination, pallor, tachypnoea and tachycardia were common. Cases 1–3 presented with regenerative anaemia, but the anaemia was initially hyporegenerative in Case 4. Other clinicopathologic abnormalities that have been documented in *Cytauxzoon* sp. infected cats, including leukocytosis, thrombocytopenia and hyperbilirubinaemia were inconsistently or not present in Cases 1–4 [24, 30, 31, 41].

In Case 4, *Cytauxzoon* sp. infection was likely the result of blood transfusion from the asymptomatic infected donor cat (Case 5). Although a false negative PCR result at first presentation cannot be ruled out, this seems unlikely given that the blood donor tested PCR-positive for *Cytauxzoon* sp. This is the first description of *Cytauxzoon* sp. transmission by blood transfusion. For *C. felis,* blood transfusion has been shown to induce severe disease if blood is collected during the schizogenous phase of disease, whereas transfusion from subclinical cats does not induce schizogony or clinical disease but leads to chronic parasitaemia in the recipient cat [66]. Because the pathogenesis of *Cytauxzoon* sp. seems to differ from *C. felis*, it is unclear whether transfusion of blood from *Cytauxzoon* sp. infected donor cats can induce clinical disease in recipients.

Case 5 of the present report showed no clinical signs of disease and no alterations in the complete blood count. This cat was used as a blood donor, given unremarkable findings of the clinical examination and negative serologic tests for FeLV and FIV. Screening potential donors for *Cytauxzoon* sp. infection was not considered necessary, as this infection had not been previously documented in Switzerland. The consensus statements of the European Advisory Board on Cat Disease (ABCD) and the American College of Veterinary Internal Medicine (ACVIM) recommend that in endemic areas, only PCR-negative cats should be used as donors [67, 68]. Given the long-term chronic parasitaemia in most *Cytauxzoon* sp. infected cats and severe disease reported in some infected cats, *Cytauxzoon* sp. should be included in the screening of all blood donor cats in central and southern Europe. Such screening should be done using PCR as blood smear evaluation is poor sensitive to detect low-grade parasitaemia.

Cases 1–3 in this study recovered following treatment with atovaquone, azithromycin and supportive care. Treatment with atovaquone (15 mg/kg PO q8h) and azithromycin (10 mg/kg PO q24h) for 10 days is regarded the treatment of choice for acute cytauxzoonosis caused by *C. felis* and has been shown to be superior to treatment with imidocarb diproprionate (3.5 mg/kg IM twice 7 days apart) [69]. Previously published *Cytauxzoon* sp. cases in Europe were treated with a variety of antiprozotoal drugs [24, 30, 31, 41], but the optimal treatment strategy for this infection remains unclear given the lack of controlled studies.

Despite clinical recovery, chronic asymptomatic parasitaemia was observed in Cases 1–3 even after a second course of treatment with atovaquone and azithromycin. Similarly, asymptomatic parasitaemia for up to 1.3 years has been documented in domestic cats in Europe [24, 31, 41]. The extent to which asymptomatic cats with chronic *Cytauxzoon* sp. infection may act as a reservoir is unclear. For *C. felis*, transmission of the agent from chronically infected domestic cats to naive cats *via* tick bites has been documented [3, 70]. This suggests that cats are competent reservoirs for *C. felis*. Furthermore, a high prevalence of chronic *C. felis* infection has recently been documented in an enzootic area in the USA [9]. The authors concluded that, because domestic cats are more likely to live near other domestic cats than near bobcats, these chronically infected cats may play an important role in disease transmission. The extent to which this may also be the case for *Cytauxzoon* sp. infection remains to be elucidated. However, indoor housing and ectoparasite control should be considered for cats chronically infected with *Cytauxzoon* sp.

In contrast to Cases 1–3, Case 4 seemed to have cleared erythroparasitaemia when it presented for a relapse of anaemia in week 38. Although to the best of our knowledge, this has not yet been reported for *C. felis* infection, clearance of *Cytauxzoon* sp. infection was recently documented in a cat in Italy that remained PCR-negative for 175 days following treatment with doxycyline and imidocarb diproprionate [41]. Although treatment with atovaquone and azithromycin may have contributed to clearing erythroparasitaemia in Case 4, this is not clear because *Cytauxzoon* sp. was still detected by PCR immediately following treatment.

## Conclusions

The present study further expands the geographical range of *Cytauxzoon* sp. infection in domestic cats in Europe and describes the accidental transmission of *Cytauxzoon* sp. by blood transfusion. The report further emphasizes that *Cytauxzoon* sp. infection should be considered in domestic cats in central Europe that are presented with anaemia. The pathogenesis and route of transmission of *Cytauxzoon* sp. requires further investigation. Given the long-term asymptomatic parasitaemia, domestic cats may represent an important reservoir for *Cytauxzoon* sp. even in areas in which no wild reservoirs are present, and healthy blood donor cats should be screened for this agent by PCR.

## Abbreviations

Ct: cycle threshold
DNA: deoxyribonucleic acid
dNTP: deoxyribonucleotide triphosphate
EDTA: ethylenediaminetetraacetic acid
FeLV: feline leukaemia virus
FIV: feline immunodeficiency virus
PCR: polymerase chain reaction
PCV: packed cell volume
PO: *per os*
rRNA: ribosomal ribonucleic acid
TNA: total nucleic acid
